# Supplementary material for: Childhood Mild Traumatic Brain Injury is Reliably Associated with Anxiety but Not Other Examined Psychiatric Outcomes at Two‐Year Follow‐up, After Adjusting for Prior Mental Health
Source: J Child Psychol Psychiatry. 2025 Jul 15;66(12):1849–59. doi: 10.1111/jcpp.70013 (PMC12626180; doi:10.1111/jcpp.70013)
Supplement: Supplementary file 1 — Table S1. Demographics and descriptive statistics for the 2‐year follow‐up sample. Table S2. Association between mental health symptoms at age 9–10 and lifetime mTBI/orthopaedic injury. Table S3. Proportion and percentage of psychiatric service use across injury groups at baseline and 2‐year follow‐up. Table S4. Association between mental health symptoms at age 11–12 and new mTBI/orthopaedic injury in the previous 24 months. Figure S1. Visualisation of missing data for psychiatric disorders and symptoms at baseline. Figure S2. Visualisation of missing data for psychiatric service use at baseline. Figure S3. Visualisation of missing data for psychiatric disorders and symptoms at 2‐year follow‐up. Figure S4. Visualisation of missing data for psychiatric service use at 2‐year follow‐up. [file JCPP-66-1849-s001.docx]

Supplementary material for

**Specific Association with Anxiety and New Childhood Mild Traumatic Brain Injury at Two-Year Follow-up but Not Other Examined Psychiatric Outcomes After Controlling for Pre-Existing Mental Health**

|  | *mTBI*  *N = 217* | *Ortho*  *N = 466* | *No Injury*  *N = 10,533* | |
| --- | --- | --- | --- | --- |
| *Sex* |  |  |  | |
| Female | 87 (40%) | 221 (47%) | 5,040 (48%) | |
| Male | 130 (60%) | 245 (53%) | 5,492 (52%) | |
| Unknown | 0 | 0 | 1 | |
| *Age* | 12.08 (11.50, 12.50) | 12.00 (11.42, 12.58) | 12.00 (11.42, 12.58) | |
| Unknown | 4 | 28 | 1,007 | |
| *Race and Ethnicity* |  |  |  | |
| Asian | 3 (1.4%) | 7 (1.5%) | 231 (2.2%) | |
| Black | 17 (7.8%) | 32 (6.9%) | 1,547 (15%) | |
| Hispanic | 30 (14%) | 80 (17%) | 2,111 (20%) | |
| Other | 29 (13%) | 45 (9.7%) | 1,098 (10%) | |
| White | 138 (64%) | 302 (65%) | 5,544 (53%) | |
| Unknown | 0 | 0 | 2 | |
| *Combined annual household income* | | | | |
| [<50K] | 35 (17%) | 85 (20%) | | 2,812 (29%) |
| [>=50K & <100K] | 67 (32%) | 129 (30%) | | 2,741 (28%) |
| [>=100K] | 105 (51%) | 216 (50%) | | 4,110 (43%) |
| Unknown | 10 | 36 | | 870 |
| *Neighbourhood safety* | | | |  |
| Strongly Disagree | 5 (2.3%) | 9 (1.9%) | | 370 (3.5%) |
| Disagree | 13 (6.0%) | 23 (4.9%) | | 703 (6.7%) |
| Neutral | 32 (15%) | 67 (14%) | | 1,770 (17%) |
| Agree | 74 (34%) | 139 (30%) | | 3,060 (29%) |
| Strongly Agree (safe) | 93 (43%) | 228 (49%) | | 4,614 (44%) |
| Unknown | 0 | 0 | | 16 |
| *Family conflict scale* | | | |  |
| 0 | 146 (67%) | 283 (61%) | | 6,554 (62%) |
| 1 | 40 (18%) | 109 (23%) | | 2,274 (22%) |
| 2 | 23 (11%) | 53 (11%) | | 1,209 (11%) |
| 3 | 8 (3.7%) | 20 (4.3%) | | 480 (4.6%) |
| Unknown | 0 | 1 | | 16 |
| *Traumatic events* | |  | |  |
| No | 129 (61%) | 281 (62%) | | 6,618 (64%) |
| Yes | 84 (39%) | 171 (38%) | | 3,667 (36%) |
| Unknown | 4 | 14 | | 248 |

**Supplementary Table 1.** Demographics and descriptive statistics for the two-year follow-up sample

^1^n (%); Median (IQR)

|  | *Unadjusted*  *Beta (95% CI)* | *Model 1*  *Beta (95% CI)* | *Model 2*  *Beta (95% CI)* |
| --- | --- | --- | --- |
| *Internalising symptoms score* |  |  |  |
| mTBI vs uninjured | 2.20 (1.70 – 2.70) | 2.23 (1.72 – 2.74) | 1.30 (0.87 – 1.80) |
| Ortho injury vs uninjured | 0.17 (-0.12 – 0.46) | 0.16 (-0.13 – 0.45) | -0.12 (-0.37 – 0.13) |
| *Externalising symptoms score* |  |  |  |
| mTBI vs uninjured | 2.20 (1.70 – 2.80) | 2.26 (1.72 – 2.80) | 1.40 (0.93 – 1.90) |
| Ortho injury vs uninjured | 0.00 (-0.31 – 0.30) | 0.13 (-0.17 – 0.44) | -0.12 (-0.39 – 0.15) |
| *Anxiety symptoms score* |  |  |  |
| mTBI vs uninjured | 0.74 (0.51 – 0.97) | 0.75 (0.52 – 0.97) | 0.39 (0.19 – 0.59) |
| Ortho injury vs uninjured | 0.06 (-0.06 – 0.19) | 0.06 (-0.07 – 0.18) | -0.05 (-0.17 – 0.06) |
| *Depression symptoms score* |  |  |  |
| mTBI vs uninjured | 0.77 (0.58 – 0.96) | 0.76 (0.57 – 0.95) | 0.47 (0.30 – 0.64) |
| Ortho injury vs uninjured | 0.01 (-0.10 – 0.11) | 0.01 (-0.09 – 0.12) | -0.07 (-0.17 – 0.02) |
| *ADHD symptoms score* |  |  |  |
| mTBI vs uninjured | 1.20 (0.88 – 1.40) | 1.10 (0.86 – 1.40) | 0.73 (0.48 – 0.98) |
| Ortho injury vs uninjured | -0.01 (-0.17 – 0.14) | 0.04 (-0.12 – 0.19) | -0.08 (-0.22 – 0.06) |
| *Conduct problems score* |  |  |  |
| mTBI vs uninjured | 0.73 (0.51 – 0.95) | 0.77 (0.55 – 0.98) | 0.49 (0.29 – 0.70) |
| Ortho injury vs uninjured | -0.04 (-0.16 – 0.08) | 0.04 (-0.08 – 0.16) | -0.04 (-0.15 – 0.07) |
| *ODD symptoms score* |  |  |  |
| mTBI vs uninjured | 0.70 (0.51 – 0.89) | 0.67 (0.49 – 0.86) | 0.40 (0.23 – 0.58) |
| Ortho injury vs uninjured | 0.02 (-0.08 – 0.13) | 0.03 (-0.08 – 0.14) | -0.05 (-0.15 – 0.05) |

**Supplementary Table 2.** Association between mental health symptoms at age 9-10 and lifetime mTBI / orthopaedic injury. TBI = traumatic brain injury. Ortho = non-TBI orthopaedic injury.

| *Baseline* | *mTBI*  *N = 450* | *Ortho*  *N = 1,604* | *No Injury*  *N = 9,808* |
| --- | --- | --- | --- |
| Psychotherapy | 36 (8.0%) | 75 (4.7%) | 440 (4.5%) |
| Medication for mental health | 36 (8.0%) | 50 (3.1%) | 391 (4.0%) |
| Outpatient support | 83 (18%) | 139 (8.7%) | 814 (8.3%) |
| Inpatient support | 4 (0.9%) | 5 (0.3%) | 51 (0.5%) |
| Any mental health service use | 124 (28%) | 246 (15%) | 1,505 (15%) |
| *Two-year follow-up* | *mTBI*  *N = 217* | *Ortho*  *N = 466* | *No Injury*  *N = 10,533* |
| Psychotherapy | 18 (8.5%) | 27 (6.2%) | 410 (4.3%) |
| Medication for mental health | 17 (8.0%) | 20 (4.6%) | 455 (4.8%) |
| Outpatient support | 27 (13%) | 44 (10%) | 789 (8.3%) |
| Inpatient support | 0 (0.0%) | 1 (0.2%) | 50 (0.5%) |
| Any mental health service use | 48 (23%) | 85 (19%) | 1,383 (15%) |

**Supplementary Table 3.** Proportion and percentage of psychiatric service use across injury groups at baseline and two-year follow-up.

|  | *Unadjusted*  *Beta (95% CI)* | *Model 1*  *Beta (95% CI)* | *Model 2*  *Beta (95% CI)* | *PSM Model*  *Beta (95% CI)* |
| --- | --- | --- | --- | --- |
| *Internalising symptoms score* |  |  |  |  |
| mTBI vs uninjured | 1.00 (0.40 – 1.70) | 1.03 (0.40 – 1.66) | 1.10 (0.46 – 1.60) | 0.74 (-0.12 – 1.6) |
| Ortho injury vs uninjured | 0.48 (0.05 – 0.92) | 0.44 (0.00 – 0.87) | 0.37 (-0.04 – 0.77) | 0.43 (-0.09 – 0.95) |
| mTBI vs ortho injury |  |  |  | 0.52 (-0.35 – 1.40) |
| *Externalising symptoms score* |  |  |  |  |
| mTBI vs uninjured | 0.75 (0.13 – 1.4) | 0.80 (0.18 – 1.47) | 0.84 (0.25 – 1.40) | 0.64 (-0.15 – 1.4) |
| Ortho injury vs uninjured | 0.27 (-0.15 – 0.70) | 0.33 (-0.10 – 0.75) | 0.24 (-0.16 – 0.65) | 0.00 (-0.54 – 0.53) |
| mTBI vs ortho injury |  |  |  | 0.65 (-0.14 – 1.4) |
| *Anxiety symptoms score* |  |  |  |  |
| mTBI vs uninjured | 0.53 (0.26 – 0.79) | 0.53 (0.27 – 0.79) | 0.53 (0.28 – 0.79) | 0.46 (0.11 – 0.80) |
| Ortho injury vs uninjured | 0.15 (-0.03 – 0.33) | 0.12 (-0.06 – 0.30) | 0.10 (-0.07 – 0.27) | 0.05 (-0.17 – 0.27) |
| mTBI vs ortho injury |  |  |  | 0.38 (0.02 – 0.74) |
| *Depression symptoms score* |  |  |  |  |
| mTBI vs uninjured | 0.25 (-0.01 – 0.50) | 0.24 (-0.02 – 0.49) | 0.25 (0.01 – 0.49) | 0.29 (-0.03 – 0.62) |
| Ortho injury vs uninjured | 0.13 (-0.04 – 0.30) | 0.11 (-0.06 – 0.29) | 0.09 (-0.08 – 0.25) | 0.04 (-0.19 – 0.26) |
| mTBI vs ortho injury |  |  |  | 0.12 (-0.21 – 0.45) |
| *ADHD symptoms score* |  |  |  |  |
| mTBI vs uninjured | 0.34 (0.02 – 0.65) | 0.30 (-0.01 – 0.61) | 0.32 (0.02 – 0.61) | 0.40 (-0.01 – 0.80) |
| Ortho injury vs uninjured | -0.01 (-0.23 – 0.20) | 0.00 (-0.21 – 0.21) | -0.04 (-0.24 – 0.17) | -0.10 (-0.37 – 0.17) |
| mTBI vs ortho injury |  |  |  | 0.38 (-0.04 – 0.80) |
| *Conduct problems score* |  |  |  |  |
| mTBI vs uninjured | 0.23 (-0.02 – 0.48) | 0.26 (0.02 – 0.51) | 0.27 (0.03 – 0.51) | 0.24 (-0.09 – 0.58) |
| Ortho injury vs uninjured | 0.12 (-0.06 – 0.29) | 0.15 (-0.02 – 0.32) | 0.13 (-0.04 – 0.29) | 0.15 (-0.05 – 0.35) |
| mTBI vs ortho injury |  |  |  | 0.19 (-0.13 – 0.51) |
| *ODD symptoms score* |  |  |  |  |
| mTBI vs uninjured | 0.16 (-0.06 – 0.39) | 0.16 (-0.07 – 0.38) | 0.17 (-0.04 – 0.38) | 0.18 (-0.10 – 0.46) |
| Ortho injury vs uninjured | 0.06 (-0.10 – 0.21) | 0.07 (-0.09 – 0.21) | 0.03 (-0.11 – 0.18) | -0.04 (-0.23 – 0.15) |
| mTBI vs ortho injury |  |  |  | 0.12 (-0.18 – 0.42) |

**Supplementary Table 4.** Association between mental health symptoms at age 11-12 and new mTBI / orthopaedic injury in the previous 24 months. TBI = traumatic brain injury. Ortho = non-TBI orthopaedic injury. PSM = propensity score matched.

**
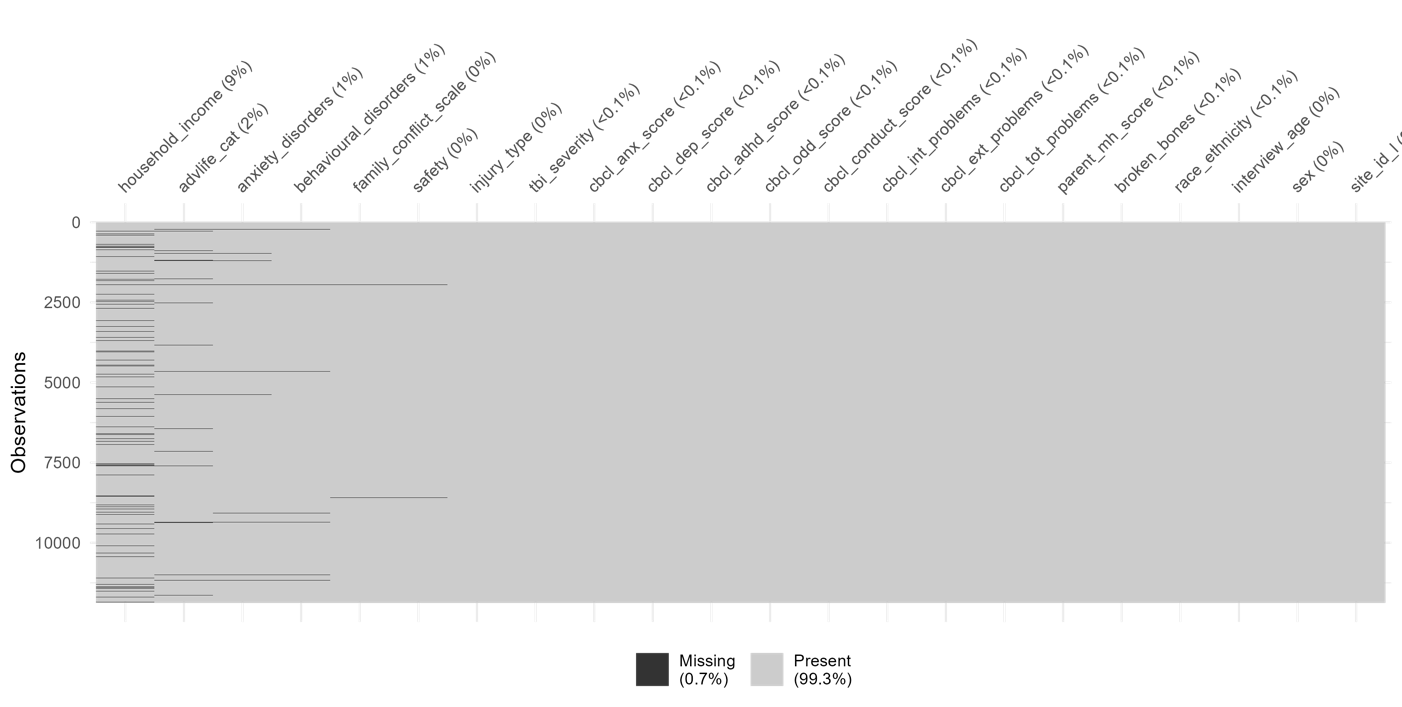
**

**Supplementary Figure 1.** Visualisation of missing data for psychiatric disorders and symptoms at baseline

**
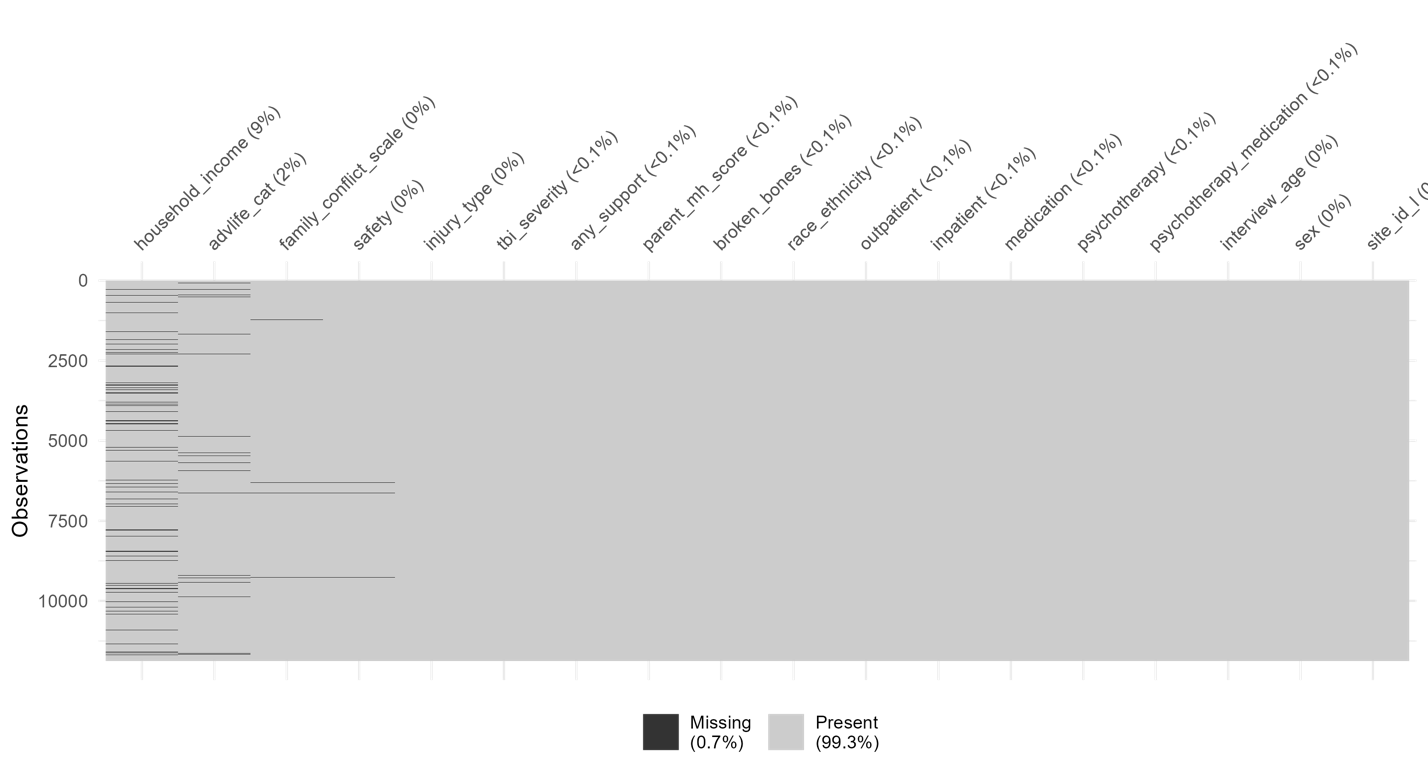
**

**Supplementary Figure 2.** Visualisation of missing data for psychiatric service use at baseline


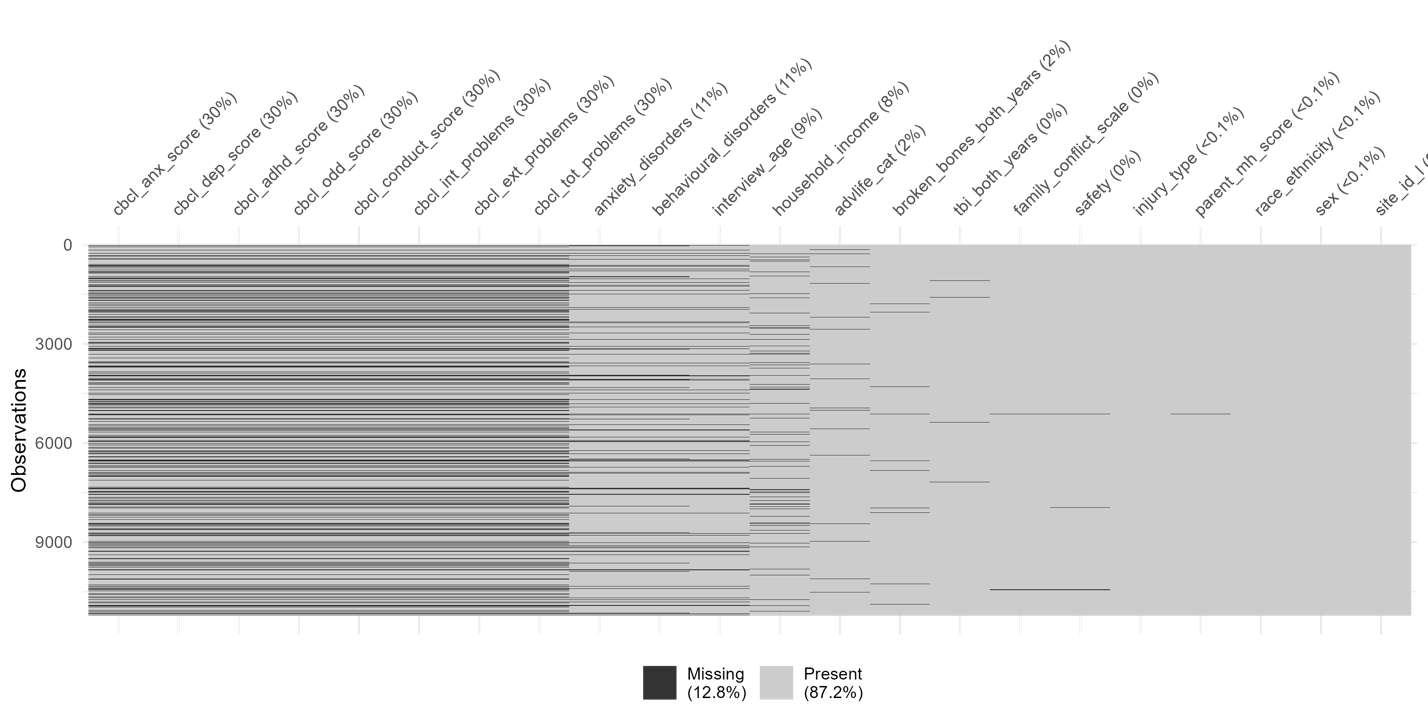


**Supplementary Figure 3.** Visualisation of missing data for psychiatric disorders and symptoms at two-year follow-up

**
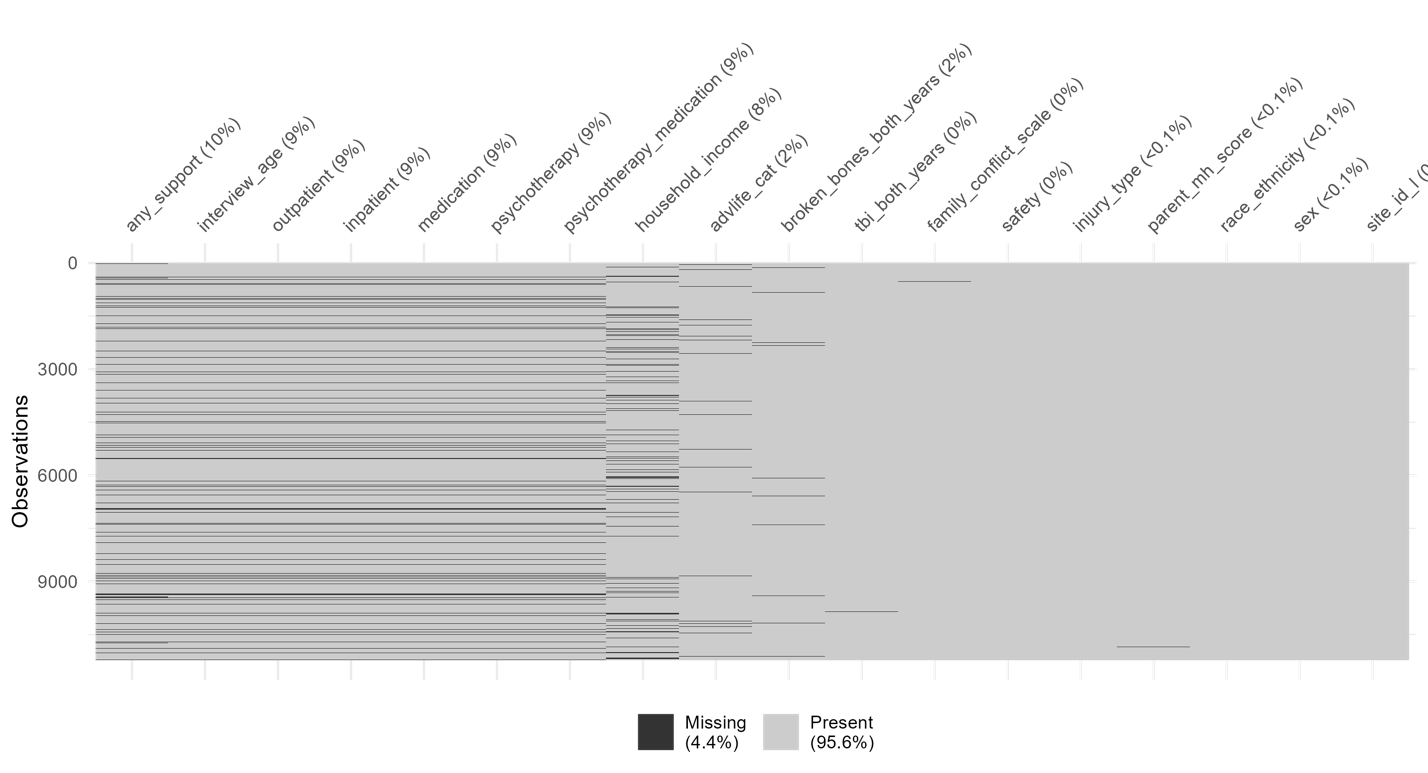
**

**Supplementary Figure 4.** Visualisation of missing data for psychiatric service use at two-year follow-up
